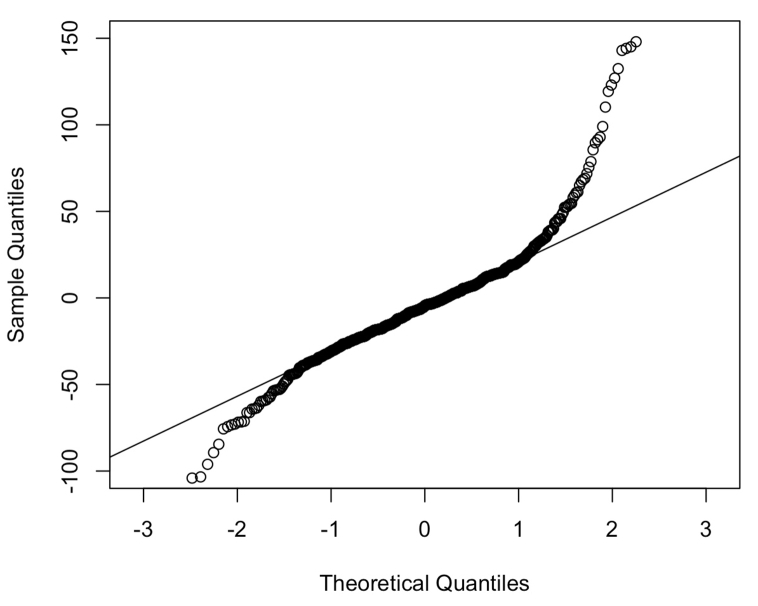# The association of depression and anxiety with postoperative opioid use following total joint arthroplasty

**DOI:** 10.1016/j.heliyon.2023.e18813

**Published:** 2023-07-29

**Authors:** Crystal Diei, Soraya Mehdipour, Pelle V. Wall, Rodney A. Gabriel

**Affiliations:** a Division of Perioperative Informatics, Department of Anesthesiology, University of California, San Diego, La Jolla, CA, USA; b School of Medicine, Department of Anesthesiology, University of California, San Diego, La Jolla, CA, USA; c Department of Biomedical Informatics, Department of Anesthesiology, University of California, San Diego, La Jolla, CA, USA

**Keywords:** Depression, Total joint arthroplasty, Opioids

## Abstract

**Background:**

The devastating opioid epidemic in the United States has been exacerbated by health care practices as well as underlying individual factors. Total joint arthroplasty (TJA) is one of the most common surgical procedures performed annually and patients frequently require opioids for pain control. Patient anxiety and depression has been shown to be associated with increased pain and poorer outcomes after TJA. Our study sought to determine if there was an association between depression/anxiety and postoperative opioid use following TJA.

**Methods:**

In this retrospective cohort study, postoperative outcomes after TJA were compared among three cohorts of patients: 1) no depression; 2) mild depression; or 3) moderate or severe depression at our institution from 2017 to 2019. Our primary outcome was persistent opioid use ≥3 months after surgery. Secondary outcomes included postoperative day 1 opioid consumption and hospital length of stay (LOS). Multivariable regression modeling was performed to control for various potential confounders.

**Results:**

Of the 542 total patients that met inclusion criteria for this study, 53 (9.8%) had mild depression and 67 (12.4%) had moderate or severe depression. Persistent opioid use ≥3 months after surgery was found in 132 (24.3%) patients. Mild depression was associated with increased odds of persistent opioid use (odds ratio 4.11, 95% confidence interval 1.65–10.18, P = 0.002). Depression was not associated with immediate postoperative opioid use or hospital LOS.

**Conclusion:**

Mild depression was associated with persistent opioid use after surgery. Future studies should investigate if better management of this comorbidity could improve outcomes in patients undergoing joint arthroplasty.

## 1 Introduction

Total hip arthroplasty (THA) and total knee arthroplasty (TKA) are two of the most common surgical procedures performed annually and opioids are regularly prescribed to treat postoperative THA and TKA pain [1]. The factors that may predispose patients to prolonged postoperative use remain under investigation.

Mental health is one factor that has been studied as a risk factor for postoperative pain and prolonged postoperative opioid use. A high prevalence of depression and anxiety symptoms in TKA and THA patients has been described preoperatively [2]. One meta-analysis found that patients with poorer preoperative psychological health showed higher postoperative pain scores and lower functional outcomes following TKA [3]. Another systematic-review found that preoperative anxiety or depression was a significant predictor of postoperative pain after TKA in all seven studies that specifically examined these states, and that patients with anxiety, depression, or lower mental health scores showed higher pain scores at baseline and at all postoperative timepoints [4]. Goesling and colleagues found that depression, catastrophizing, and worse pain and functioning were consistent with persistent opioid use 6 months following TKA and THA [5]. One prospective study of patients undergoing primary THA found depression was correlated with increased opioid use and longer duration of opioid use postoperatively [6]. Several additional studies have shown depression and anxiety can be strong predictors of postoperative opioid use after arthroplasty surgeries [[7], [8], [9], [10]]. However, other studies have failed to identify a statistically significant association between depression, anxiety, and postoperative opioid use in TKA and THA patients [11,12].

Further investigation is needed to assess the association between depression, its severity, and associated anti-depressant medications with postoperative opioid use following joint arthroplasty. Identifying risk factors that may predispose patients to persistent postoperative opioid use would allow for greater clinical caution and additional safeguards in certain surgical patients to better prevent opioid misuse and the harm that often follows. The hypothesis of the study was that depression would be associated with greater postoperative opioid consumption and persistent postoperative opioid use.

## 2 Methods

### 2.1 Study sample

This retrospective study was approved by our institution’s Human Research Protections Program for the collection of data from our electronic medical record system, and the informed consent requirement was waived (protocol approval #191006). Data were manually collected retrospectively from the electronic medical record system from our institution. Data from all patients that underwent elective hip or knee arthroplasty from 2017 to 2019 were extracted. Cases that were deemed emergent, bilateral surgery, or involved non-joint arthroplasty surgery (e.g. open reduction and internal fixation of femoral fracture) were excluded from the analysis.

Patients undergoing joint arthroplasty at our institution receive preoperative acetaminophen and celecoxib. For knee arthroplasty, adductor canal peripheral nerve blocks with perineural catheters were offered. No peripheral nerve blocks were performed for patients undergoing hip arthroplasty. Intraoperatively, neuraxial anesthesia was the preferred anesthetic, however, general anesthesia was performed due to patient preference, contraindications to neuraxial anesthesia, or technical difficulties with neuraxial placement. The orthopedic surgeon performed periarticular injections intraoperatively prior to closure. Postoperatively, patients received a standard regimen of oral opioids (including oxycodone and tramadol) and acetaminophen.

### 2.2 Primary objective and data collection

The objective of this study was to determine if there was an association between depression and postoperative opioid use after joint arthroplasty. The presence and severity of depression and anxiety was ascertained from the preoperative anesthesia history and physical note from day of surgery. This note was created by the anesthesiology provider who took care of the patient on day of surgery. Thus, the health information recorded in this note is the most up-to-date history for the patient ascertained and confirmed by the anesthesia provider assigned to that case. Only those that were described to have an active history of depression or anxiety were labeled as such. The limitation of this retrospective approach is that some patients may be classified as not having depression/anxiety when they indeed have a history. Nonetheless, we also captured outpatient medication usage related to depression and/or anxiety. The study population was divided into three cohorts based on the severity of depression ascertained from the anesthesia preoperative note: 1) no depression; 2) mild severity of depression; and 3) >mild severity of depression (e.g. moderate or severe). The primary outcome of interest was persistent opioid use, defined as continued opioid use ≥3 months after surgery (this was defined as a binary outcome and was ascertained via manual chart review from orthopedic surgery follow-up clinic notes). Secondary outcomes of interest were total opioid consumption on postoperative day (POD) 1 and hospital length of stay (LOS). The primary exposure was diagnosis of depression and/or anxiety. Opioid consumption was measured in oxycodone equivalents (OEQs). In addition, the following confounding variables were collected: active history of anxiety, joint involved during surgery (e.g. hip versus knee), age, body mass index, psychiatric medications, preoperative opioid use, obstructive sleep apnea, diabetes mellitus, chronic renal insufficiency, alcohol use, active smoking, marijuana use, and perioperative anesthesia details (including use of peripheral nerve block and primary anesthesia type [neuraxial versus general anesthesia]). Information regarding psychiatric medications was obtained from the anesthesia preoperative note, which listed active medications for that patient at the time of surgery. The categories of medications for the analysis were benzodiazepines (e.g. alprazolam, clonazepam, lorazepam), selective serotonin reuptake inhibitors (SSRI, e.g. fluoxetine, paroxetine, escitalopram), anti-psychotics (e.g. olanzapine, quetiapine, aripiprazole), serotonin-norepinephrine reuptake inhibitors (SNRI, e.g. venlafaxine, desvenlafaxine), and norepinephrine and dopamine reuptake inhibitors (NDRI, e.g. bupropion).

### 2.3 Statistical analysis

Statistical analysis was conducted using R (Version 4.0.4) Statistical Programming Language. The study population was divided into three cohorts based on the severity of depression ascertained from the anesthesia preoperative note: 1) no depression; 2) mild severity of depression; and 3) >mild severity of depression (e.g. moderate or severe). Analysis of variance (ANOVA) and chi-square test was used to compare continuous and categorical variables, respectively, among the three cohorts. To model the association between depression and persistent opioid use, we performed multivariable logistic regression (using the “glm” function in R) and controlled for potential confounder variables described above. The odds ratio (OR) and 95% confidence intervals (CI) were reported for each covariate included in the model. To model the association between depression and POD1 opioid consumption, we performed multivariable linear regression (using the “lm” function in R) and controlled for potential confounder variables described above. The coefficient estimate and standard error were reported for each covariate included in this model. To assess whether the residuals of this multivariable linear regression model were normally distributed, we generated its corresponding Q-Q plot (using the “qqnorm” function in R). Finally, to model the association of depression with hospital length of stay, a multivariable Poisson regression (using the “glm” function in R) was performed and the relative risk (RR) and 95% CI were reported. To quantify degree of overdispersion in the Poisson model, we calculated its dispersion parameter and its P-value using the “AER” R Package (function “dispersiontest”). A P-value of <0.05 was considered statistically significant.

## 3 Results

Of the 542 total patients that met inclusion criteria for this study, 53 (9.8%) had mild depression and 67 (12.4%) had moderate or severe depression. There were 69 (12.7%) patients with an active history of anxiety. Persistent opioid use ≥3 months after surgery was found in 132 (24.3%) patients (Table 1). On unadjusted analysis, patients with mild depression had the highest rate of persistent opioid use (43.4%) compared to those with moderate/severe depression (28.4%) and no depression (21.3%) (P < 0.001). Patients with moderate/severe depression had the highest mean POD1 opioid use (71.3 mg) compared to those with mild depression (47.0 mg) and no depression (32.0 mg) (P < 0.001). Patients with mild depression had the longest mean hospital length of stay (3.4 days) compared to those with moderate/severe depression (3.0 days) and no depression (2.7 days) (P < 0.001).

**Table 1:** Baseline characteristics of three surgical cohorts: 1) no depression, 2) mild depression, or 3) >mild severity depression.

		No Depression		Mild Depression		Moderate or Severe Depression		
		n	%	n	%	n	%	P-value
Total		422		53		67		
Persistent Opioid Use		90	21.3	23	43.4	19	28.4	***0.012***
POD1 Opioid Use (OEQ mg), mean (SD)		32.0 (50.1)		47.0 (44.8)		71.3 (107.4)		***<0.001***
Length of Stay, mean (SD)		2.7 (3.1)		3.4 (3.2)		3.03 (1.9)		***<0.001***
Anxiety		28	6.6	19	35.8	22	32.8	***<0.001***
Medications								
	Benzodiazepine	10	2.4	16	30.2	15	22.4	***<0.001***
	SSRI	2	0.5	24	45.3	38	56.7	***<0.001***
	Anti-Psychotic	0	0.0	3	5.7	16	23.9	***<0.001***
	SNRI	2	0.5	10	18.9	12	17.9	***<0.001***
	NDRI	0	0.0	0	0.0	21	31.3	***<0.001***
Surgical Procedure								0.921
	Knee Arthroplasty	212	50.2	28	52.8	33	49.3	
	Hip Arthroplasty	210	49.8	25	47.2	34	50.7	
Age (years), mean (SD)		68.6 (11.2)		68.8 (9.1)		64.4 (9.2)		0.112
Male Sex		158	37.4	13	24.5	13	19.4	***0.005***
Body Mass Index (kg/m2), mean (SD)		28.9 (4.9)		28.5 (5.2)		31.1 (6.9)		0.093
Smoking History		93	22.0	20	37.7	23	34.3	***0.008***
Alcohol Use		211	50.0	27	50.9	25	37.3	0.144
Renal Disease		27	6.4	5	9.4	6	9.0	0.999
Obstructive Sleep Apnea		73	17.3	9	17.0	12	17.9	0.611
Marijuana Use		21	5.0	1	1.9	4	6.0	0.546
Peripheral Nerve Block		197	46.7	27	50.9	30	44.8	0.792
Neuraxial Anesthesia		260	61.6	23	43.4	24	35.8	***<0.001***

Categorical and continuous variables were compared via chi-square test and ANOVA, respectively.

*Abbreviations*: NDRI, norepinephrine and dopamine reuptake inhibitor; OEQ, oxycodone equivalents; POD1, postoperative day 1; SD, standard deviation; SNRI, serotonin-norepinephrine reuptake inhibitor; SSRI, selective serotonin reuptake inhibitor.

Next, a multivariable logistic regression was performed to control for various confounders and model the association of depression and persistent opioid use (Table 2). Compared to those without depression, only mild depression (OR 4.11, 95% CI 1.65–10.18, P = 0.002) had statistically significant 4.11-fold increased odds for persistent opioid use. An active history of anxiety (P = 0.36) did not show statistical significance for persistent opioid use. Use of psychiatric medications also did not demonstrate statistically significant associations with persistent opioid use (all P > 0.05). Age had a 21% decreased odds (in decades, OR 0.79, 95% CI 0.64–0.96, P = 0.021), smoking history had 68% decreased odds (OR 0.32, 95% CI 0.17–0.61, P = 0.005), and marijuana use had 4.4-fold increased odds (OR 4.39, 95% CI 1.80–10.69, P = 0.001) for persistent opioid use.

**Table 2:** Results of multivariable logistic regression modeling persistent opioid use after joint arthroplasty.

Covariate		OR (95% CI)	P-value
Depression			
	None	Reference	
	Mild	4.11 (1.65, 10.18)	***0.002***
	Moderate/Severe	2.11 (0.63, 7.08)	0.232
Anxiety		1.56 (0.61, 3.98)	0.361
Medications			
	Benzodiazepine	0.48 (0.14, 1.68)	0.254
	SSRI	0.44 (0.15, 1.25)	0.126
	Anti-Psychotic	0.47 (0.10, 2.23)	0.347
	SNRI	1.30 (0.40, 4.21)	0.662
	NDRI	0.48 (0.09, 1.82)	0.231
Surgical Procedure			
	Knee Arthroplasty	Reference	
	Hip Arthroplasty	1.60 (0.44, 5.86)	0.483
Age (decades)		0.79 (0.64, 0.96)	***0.021***
Male Sex		0.80 (0.48, 1.33)	0.634
Body Mass Index (kg/m2)		1.01 (0.96, 1.06)	0.544
Smoking History		0.32 (0.17, 0.61)	***<0.001***
Alcohol Use		0.67 (0.42, 1.09)	0.112
Renal Disease		1.01 (0.39, 2.61)	0.988
Obstructive Sleep Apnea		1.49 (0.83, 2.67)	0.189
Marijuana Use		4.39 (1.80, 10.69)	***0.001***
Peripheral Nerve Block		2.47 (0.69, 8.87)	0.162
Neuraxial Anesthesia		0.93 (0.58, 1.50)	0.774

*Abbreviations*: CI, confidence interval; NDRI, norepinephrine and dopamine reuptake inhibitor; OR, odds ratio; SNRI, serotonin-norepinephrine reuptake inhibitor; SSRI, selective serotonin reuptake inhibitor.

To model the association of depression and POD1 opioid use, a multivariable linear regression was implemented to model and control for multiple confounders (Table 3). Normality of the model’s residuals are visually represented in its corresponding Q-Q plot (Supplementary Fig. 1). Neither depression nor anxiety demonstrated statistically significant associations with opioid use on POD1 (all P > 0.05). Patients that had an active history of using benzodiazepines (estimate = 48.08 mg, 95% CI 22.54–73.62, P < 0.001) or an NDRI (estimate = 76.7 mg, 95% CI 46.55–105.31, P < 0.001) as part of their home regimen used more opioids, while those on an SNRI (estimate = −36.08 mg, 95% CI-63.64 to −8.52, P = 0.012) used less.

**Table 3:** Results of multivariable linear regression modeling opioid use (oxycodone equivalents) on postoperative day 1 after joint arthroplasty.

Covariate		Estimate (95% CI)	P-value
Depression			
	None	Reference	
	Mild	18.95 (−1.81, 39.71)	0.074
	Moderate/Severe	9.09 (−17.17, 35.35)	0.491
Anxiety			0.554
Medications			
	Benzodiazepine	48.08 (22.54, 73.62)	***<0.001***
	SSRI	−14.58 (−36.43, 7.27)	0.177
	Anti-Psychotic	5.99 (−24.76, 36.74)	0.713
	SNRI	−36.08 (−63.64, −8.52)	***0.012***
	NDRI	75.93 (46.55, 105.31)	***<0.001***
Surgical Procedure			
	Knee Arthroplasty	Reference	
	Hip Arthroplasty	−2.75 (−8.16, 2.66)	0.823
Age (decades)		−11.16 (−15.55, −6.77)	***<0.001***
Male Sex		−6.73 (−16.98, 3.52)	0.194
Body Mass Index (kg/m2)		0.74 (−0.22, 1.70)	0.145
Smoking History		16.44 (5.54, 27.34)	***0.003***
Alcohol Use		−0.45 (−10.27, 9.37)	0.931
Renal Disease		−1.39 (−19.79, 17.01)	0.888
Obstructive Sleep Apnea		13.06 (0.50, 25.62)	***0.041***
Marijuana Use		−0.03 (−22.67, 22.61)	0.992
Peripheral Nerve Block		14.97 (−8.84, 38.79)	0.225
Neuraxial Anesthesia		−15.63 (−25.57, −5.69)	***0.002***

*Abbreviations*: CI, confidence interval; NDRI, norepinephrine and dopamine reuptake inhibitor; SNRI, serotonin-norepinephrine reuptake inhibitor; SSRI, selective serotonin reuptake inhibitor.

Finally, a multivariable Poisson regression was performed to model depression to hospital LOS following joint arthroplasty (Table 4). Depression and anxiety did not demonstrate a statistically significant association with LOS (all P > 0.05). The use of peripheral nerve blocks (RR 0.71, 95% CI 0.56–0.89, P = 0.003) and neuraxial anesthesia as primary anesthetic for surgery (RR 0.73, 95% CI 0.56–0.89, P < 0.001) were associated with decreased LOS. For the Poisson regression model, the dispersion parameter was 1.2 (P = 0.251), which suggested minimal issues with overdispersion.

**Table 4:** Results of multivariable Poisson regression modeling hospital length of stay after joint arthroplasty.

Covariate		RR (95% CI)	P-value
Depression			
	None	Reference	
	Mild	1.08 (0.86, 1.35)	0.497
	Moderate/Severe	0.87 (0.66, 1.16)	0.364
Anxiety			0.844
Medications			
	Benzodiazepine	1.14 (0.86, 1.50)	0.362
	SSRI	1.15 (0.91, 1.45)	0.253
	Anti-Psychotic	1.18 (0.85, 1.64)	0.314
	SNRI	1.22 (0.92, 1.61)	0.162
	NDRI	1.20 (0.88, 1.63)	0.256
Surgical Procedure			
	Knee Arthroplasty	Reference	
	Hip Arthroplasty	0.62 (0.49, 0.79)	***<0.001***
Age (decades)		0.95 (0.90, 0.99)	***0.034***
Male Sex		0.88 (0.77, 0.99)	***0.041***
Body Mass Index (kg/m2)		0.99 (0.98, 1.01)	0.332
Smoking History		1.03 (0.91, 1.71)	0.633
Alcohol Use		0.86 (0.77, 0.97)	***0.015***
Renal Disease		1.41 (1.16, 1.70)	***<0.001***
Obstructive Sleep Apnea		1.18 (1.03, 1.36)	***0.024***
Marijuana Use		1.07 (0.82, 1.04)	0.613
Peripheral Nerve Block		0.71 (0.56, 0.89)	***0.003***
Neuraxial Anesthesia		0.73 (0.56, 0.89)	***<0.001***

*Abbreviations*: CI, confidence interval; NDRI, norepinephrine and dopamine reuptake inhibitor; RR, relative risk; SNRI, serotonin-norepinephrine reuptake inhibitor; SSRI, selective serotonin reuptake inhibitor.

## 4 Discussion

In this study, anxiety and depression were common in total joint arthroplasty patients, present in nearly a third of patients. Patients with mild depression were associated with 4-fold increased odds of persistent opioid use 3 months after surgery compared to patients without depression. Depression did not demonstrate statistically significant associations with acute opioid use (e.g. POD1 opioid use) or hospital LOS. An active history of anxiety also did not demonstrate statistically significant associations with any of the studied outcomes.

In one study, major depressive disorder was found to be associated with increased opioid requirements in the immediate postoperative period following total joint arthroplasty surgery [13]. We did not find an association with depression and opioid use during the immediate postoperative period. However, we did find that certain psychiatric medications (e.g. NDRI and benzodiazepines) were indeed associated with increased opioid use. This finding would suggest that it may not be the clinical diagnosis of depression that places patients higher at risk for acute opioid use, but rather the utilization of NDRIs or benzodiazepines at baseline. Kurkis et al. (2021) also found patients with depression had greater postoperative opioid consumption and that opioid use in these patients lasted longer (20.3 ± 15.6 versus 7.2 ± 7.3 days) [6]. Other studies have also identified an association between depression/anxiety and prolonged opioid use 3 months after total shoulder arthroplasty and TKA [7,9,10], and even 6 months after surgery in one study [8]. In contrast, some studies have shown no association between depression/anxiety and persistent opioid usage ≥3 months postoperatively [11,12,14]. These discrepant findings may be the result of varying ways of defining or measuring depression and anxiety in the study populations and certainly warrant further investigation given the importance of mitigating harm from opioid misuse, dependency, and addiction. Furthermore, we controlled for severity of depression as well as common psychiatric medications.

Some studies have shown significantly longer hospital stays in patients with psychological distress and depressive symptoms after total joint arthroplasty [[15], [16], [17]]. A limiting factor for patient discharge from the hospital after joint arthroplasty is adequate pain control. Depression and anxiety can also cause a lack of motivation for recovery and poor adherence to treatment regimens which may also contribute to a longer hospital course [18]. In contrast, our study demonstrated no statistically significant associations between depression or anxiety with hospital LOS. The differences seen in our study compared to others could be related to our statistical approach utilizing severity of depression and psychiatric medications as covariates to our models.

Prehabilitation is a proactive approach to perioperative care that can improve health outcomes for patients who have depression and anxiety. Treating patients' psychological disorders prior to surgery could lead to enhanced postoperative satisfaction, improved surgical outcomes, decreased postoperative pain, and shorter hospital stays, which could lead to a decreased risk of chronic postoperative opioid use. Furthermore, the use of transitional pain clinics can be leveraged to target patients identified at higher risk for postoperative persistent opioid use.

There are several limitations associated with this study. The retrospective design relies on accurate charting. All patients were taken from a single academic center, limiting the generalizability of our results. Furthermore, some patients may have had a diagnosis of depression/anxiety that was not been readily available in their electronic medical record (i.e. managed at another healthcare institution); however, the active diagnosis of depression was ascertained by the anesthesiologist on day of surgery and recorded in the preoperative note. Furthermore, we reviewed the active medication list which was also recorded in the anesthesia preoperative note day of surgery. Furthermore, due to the retrospective nature of the study, we did not have data related to patient satisfaction or provider habits for opioid prescribing postoperatively. Another limitation is that patients with persistent opioid use were identified based on chart review, which may have inadvertently excluded some patients if such information was not detailed in their orthopedic follow-up visits. Furthermore, we limited our outcome to >3 months postoperatively. Future studies should study even longer term outcomes at the 1 year mark. Finally, we investigated the association of various patient factors (e.g. medication use, anxiety, etc) with the primary and secondary outcomes and did not observe any statistically significant associations. However, this may be due the sample size not providing adequate power to detect these differences. Thus, we cannot definitively conclude lack of association for some of our variables.

As the United States is in the midst of an opioid epidemic, it is imperative to take action to fight this public health crisis. We must continue to investigate how clinicians approach postoperative pain treatment to minimize the risk that patients develop chronic opioid use. Preoperative characteristics, such as a patient’s mental health, has the potential to greatly influence surgical outcomes and can provide important insights into patient specific medical care. The use of transitional pain clinics can be leveraged to target patients with mental health disorders identified at higher risk for postoperative persistent opioid use.

## Author contribution statement

Crystal Diei; Rodney Gabriel: Conceived and designed the experiments; Performed the experiments; Analyzed and interpreted the data; Contributed reagents, materials, analysis tools or data; Wrote the paper.

Soraya Mehdipour; Pelle V. Wall: Analyzed and interpreted the data; Wrote the paper.

## Data availability statement

The authors do not have permission to share data.

## Declaration of competing interest

The authors declare the following financial interests/personal relationships which may be considered as potential competing interests: Dr. Gabriel’s institution has received funding and/or product for research purposes from Epimed, Teleflex, SPR Therapeutics, Infutronix, Precision Genetics, and Merck. Dr. Gabriel’s institution serves as consultant for Avanos, in which dr. Gabriel represents.

## Figures and Tables

**figs1:**